# George Leich

**Published:** 1896-09

**Authors:** 


VETERINARIAN GEORGE LEICH, of 1540 Gates Ave., Brooklyn, died on August 2, from blood-poisoning, following an injury to his little finger received during an operation on one of his patients.

The wife of Dr. William R. Rosekrans, graduate of the American, Class of '96, died in August.

